# FNDC5/irisin improves the therapeutic efficacy of bone marrow-derived mesenchymal stem cells for myocardial infarction

**DOI:** 10.1186/s13287-020-01746-z

**Published:** 2020-06-10

**Authors:** Jingyu Deng, Ning Zhang, Yong Wang, Chao Yang, Yabin Wang, Chao Xin, Jinming Zhao, Zhitao Jin, Feng Cao, Zheng Zhang

**Affiliations:** 1 Department of Cardiology, Postgraduate Training Base in PLA Rocket Force Characteristic Medical Center, Jinzhou Medical University, Jinzhou, 121001 Liaoning China; 2 Central Beijing Medical District, Chinese PLA General Hospital, Fuxing-Road, Haidian, Beijing, 100853 China; 3 Department of Nuclear Medicine, the Fifth Medical Center,, Chinese PLA General Hospital (Former 307th Hospital of the PLA), Beijing, 100071 China; 4 Department of Cardiology, PLA Rocket Force Characteristic Medical Center, Beijing, 100088 China; 5 National Clinical Research Center for Geriatric Diseases, Second Medical Center, Chinese PLA General Hospital, Beijing, 100853 China; 6 Department of Otorhinolaryngology Head and Neck Surgery, Beijing Tongren Hospital, Capital Medical University, Beijing, 100730 China

**Keywords:** Bone marrow mesenchymal stem cells (BM-MSCs), Myocardial infarction (MI), Fibronectin type III domain-containing protein 5 (FNDC5), Irisin, Apoptosis, Cell viability

## Abstract

**Background:**

The beneficial functions of bone marrow mesenchymal stem cells (BM-MSCs) decline with decreased cell survival, limiting their therapeutic efficacy for myocardial infarction (MI). Irisin, a novel myokine which is cleaved from its precursor fibronectin type III domain-containing protein 5 (FNDC5), is believed to be involved in a cardioprotective effect, but little was known on injured BM-MSCs and MI repair yet. Here, we investigated whether FNDC5 or irisin could improve the low viability of transplanted BM-MSCs and increase their therapeutic efficacy after MI.

**Methods:**

BM-MSCs, isolated from dual-reporter firefly luciferase and enhanced green fluorescent protein positive (Fluc^+^–eGFP^+^) transgenic mice, were exposed to normoxic condition and hypoxic stress for 12 h, 24 h, and 48 h, respectively. In addition, BM-MSCs were treated with irisin (20 nmol/L) and overexpression of FNDC5 (FNDC5-OV) in serum deprivation (H/SD) injury. Furthermore, BM-MSCs were engrafted into infarcted hearts with or without FNDC5-OV.

**Results:**

Hypoxic stress contributed to increased apoptosis, decreased cell viability, and paracrine effects of BM-MSCs while irisin or FNDC5-OV alleviated these injuries. Longitudinal in vivo bioluminescence imaging and immunofluorescence results illustrated that BM-MSCs with overexpression of FNDC5 treatment (FNDC5-MSCs) improved the survival of transplanted BM-MSCs, which ameliorated the increased apoptosis and decreased angiogenesis of BM-MSCs in vivo. Interestingly, FNDC5-OV elevated the secretion of exosomes in BM-MSCs. Furthermore, FNDC5-MSC therapy significantly reduced fibrosis and alleviated injured heart function.

**Conclusions:**

The present study indicated that irisin or FNDC5 improved BM-MSC engraftment and paracrine effects in infarcted hearts, which might provide a potential therapeutic target for MI.

## Background

The treatments focus on retaining the function of the remaining cardiomyocytes, but myocardial infarction (MI) still remains a major cause of morbidity and mortality worldwide [1, 2]. Recently, mesenchymal stem cell (MSC) transplantation has held great promise for multiple diseases, such as MI [3–6]. The illustrated underlying therapeutic mechanisms include engraftment [7], paracrine [8], and other effects, like exosomes [6]. These mechanisms play a role on the premise that the engrafted MSCs survive for a certain period. However, many studies have shown that only a small number of transplanted MSCs eventually survive to play protective effects [9–11]. Also, evidences indicated that under myocardial ischemia conditions, hypoxic stress caused increased apoptosis of MSCs and reduced its therapeutic effects [1, 12]. Therefore, it is reasonable to assume that promoting the low engrafted MSC survival in ischemia myocardial tissue may be the key to improving stem cell therapeutic efficacy after MI.

FNDC5 is a transmembrane protein that possesses two domains (fibronectin III and carboxy-terminal, respectively) located in the cytoplasm [13]. Previous study has indicated that FNDC5 can be cleaved by an unknown enzyme, and then, the extracellular domain of FNDC5 was named as irisin [14]. In addition, irisin was recognized as a myokine secreted from the skeletal muscle and heart [15, 16]. Furthermore, irisin has been considered as a multifunctional peptide which is involved in regulating cardiovascular function [13, 15, 16]. Meanwhile, accumulating studies demonstrated that irisin was involved in cardioprotective effect, such as anti-apoptosis, increased cell viability, and anti-oxidative stress by various pathways [17, 18].

However, the protective effect of FNDC5/irisin in hypoxia-induced low viability of transplanted BM-MSCs is still unclear. Therefore, we hypothesized that irisin may protect against increased apoptosis and paracrine dysfunction of BM-MSCs induced by hypoxia. Moreover, genetically modified BM-MSCs overexpressing FNDC5 may promote the functional survival of transplanted BM-MSCs and increase the cardiac protective effect.

## Methods

### Animal

Adult male C57BL/6 mice (8–12 weeks of age, 20–25 g) were provided by the PLA Rocket Force Characteristic Medical Center (Beijing, China). Meanwhile, Fluc^+^–eGFP^+^ transgenic mice (Tg(*fluc–egfp*)) were purchased from Contag Laboratory (Stanford, CA, USA). Mice were created on the C57BL/6 background to stably express both firefly luciferase (Fluc) and enhanced green fluorescence protein (eGFP) in all tissues and organs. As alleged up front [1], mice were placed into a temperature-controlled animal facility with a 12-h light/dark cycle (light cycle, 8:00 a.m. to 8:00 p.m.), with tap water and rodent chow provided ad libitum. All animal experiments were performed by a protocol approved by the Animal Care and Use Committee of the PLA Rocket Force Characteristic Medical Center (ID: 5034) and were in compliance with the Guidelines for the Care and Use of Laboratory Animals, as published by the National Academy Press.

### Isolation, culture, and treatments of BM-MSCs

BM-MSCs were isolated and expanded as described [12]. Briefly, bone marrow was flushed from the femur and tibia of adult Tg(*fluc–egfp*) mice with fetal bovine serum (FBS) (Invitrogen, Carlsbad, CA, USA)-free Dulbecco’s modified Eagle’s medium (DMEM) (Corning, Manassas, VA, USA). After passing through a 70-μm strainer and centrifugation at 1200 rpm for 5 min at room temperature, the cell pellet was re-suspended in DMEM supplemented with 20% FBS and incubated at 37 °C in an atmosphere containing 5% CO_2_. After 24 h, the medium was replaced to remove the non-adherent cells and then was completely replaced every 3 days. Third-passage BM-MSCs with optimal growth at the third generation were applied for different treatments to avoid contamination with other types of cell. Then, cells were treated with irisin (20 nmol/L) in the presence or absence of hypoxia for 48 h [17].

### FNDC5 overexpression

As described previously [19], FNDC5 cDNA was cloned from cells. The primer sequences were as follows: 5′-ATG CAC CCC GGG CCG CCC CG-3′ (forward) and 5′-GTC CCC TCT CTC CCT GAG C-3′ (reverse). The PCR product and the pIRES2-EGFP vector (from Clontech) were digested by EcoRI and BamHI restriction sites. And the FNDC5 fragment was ligated into the pIRES2-EGFP vector that was used as control. Then, Lipofectamine and plasmids were diluted in Opti-MEM (Gibco), separately. The two solutions were then mixed in a ratio of 1:1 and incubated at 20–25 °C for 5 min. Once BM-MSCs reached 60–70% fusion, the mixture was added into the cells and then incubated and processed for the indicated time.

### Hypoxia/serum deprivation injury

Hypoxia/serum deprivation (H/SD) injury was performed for the hypoxic stress of BM-MSCs as described previously [20]. Briefly, after being replaced in glucose-free DMEM without FBS, BM-MSCs were exposed to hypoxia (94% N_2_/5% CO_2_/1% O_2_) with an anaerobic system (Thermo Forma) at 37 °C for 12, 24, and 48 h, respectively. Also, BM-MSCs in the control group incubated under normoxic conditions (37 °C in 95% air, 5% CO_2_) with full medium for equivalent periods.

### Measurement of BM-MSC apoptosis

BM-MSC apoptosis was determined by flow cytometry with an Annexin V-FITC/PI Kit (Merck) according to the manufacturer’s instructions [21]. In brief, cells were re-suspended in 200 μL of binding buffer. Cells were incubated with 10 μL of Annexin V solution and 5 μL propidine iodide (PI) at room temperature for 30 min, respectively. The cells were immediately analyzed on a FACSC-LSR (Becton, Dickinson and Company, San Jose, CA). Meanwhile, the caspase-3 activity was measured using a Caspase-3 Assay kit (Clontech, MountainView, CA) according to the manufacturer’s instructions for 3 times. And these assays were performed in a blinded manner.

### Cell viability assay

The cell viability was assessed by 3-(4,5-dimethylthiazol-2-yl)-2,5-diphenyltetrazolium bromide (MTT) assay as described previously [22]. Briefly, BM-MSCs were plated in 96-well plates at 1 × 105 cells/well. After an overnight incubation, BM-MSCs with different treatments were incubated for 72 h. Then, MTT solution (Sigma) was added into a final concentration of 0.5 g/L to each well. These cells in 96-well plates were cultured in a 5% CO2 incubator at 37 °C for 4 h, and further, the medium was aspirated. Next, 200 μL dimethyl sulphoxide (DMSO) was added into each well. The absorbance was determined at a wavelength of 490 nm. In addition, optical density (OD) values of each group were detected in six duplicate wells and their averages were calculated. We did all assays blindly.

Furthermore, the viability of cells was also assessed by bioluminescence imaging (BLI) with the IVIS Kinetic system (Caliper, Hopkinton, MA, USA) [21]. In brief, BM-MSCs were plated in 24-well plates (5 × 10^4^ per well). Cells were given different treatments, and then, the medium was removed. BM-MSCs were incubated with d-luciferin reporter probe (4.5 μg/mL) and further measured by the IVIS Xenogen Kinetic system (Caliper Life Sciences, USA).

### Determination of VEGF, bFGF, IGF-1, and HGF

Enzyme-linked immunosorbent assay (ELISA) was performed to determine the concentrations of vascular endothelial growth factor (VEGF), basic fibroblast growth factor (bFGF), insulin-like growth factor (IGF)-1, and hepatocyte growth factor (HGF) secreted by BM-MSCs following the manufacturer’s instructions. All samples and standards were measured blindly for three times.

### Exosome isolation and quantification

BM-MSCs were cultured with DMEM containing 5% exosome-depleted FBS (Thermo Fisher Scientific). Subsequently, every other day, the supernatant of cultured cells was collected; centrifuged at 2000*g* for 20 min at 4 °C followed by 10,000*g* for 30 min at 4 °C in order to remove cellular debris, apoptotic bodies, and microvesicles; and then filtered with 0.22-μm filters. Isolation of exosomes was performed with ultracentrifugation at 120,000*g* for 90 min at 4 °C. The isolated exosomes were washed with 10 mL of PBS and verified by transmission electron microscopy (TEM) [23].

The concentration of exosomes was analyzed by NanoSight NS300 system equipped with a 405-nm laser [24]. Briefly, 10 μL of exosomes with 990 μL of 0.22 μm filtered sterile PBS was pushed slowly with a 1-mL syringe and illuminated by a laser. Next, they were recorded in 30-s sample videos under Brownian motion, which were analyzed with the Nanoparticle Tracking Analysis (NTA) analytical software (NanoSight, version 3.0). At least 3 videos were captured for each individual sample. The capture and analysis settings were manually set in accordance with the manufacturer’s instructions.

### Myocardial infarction model

MI was accomplished by ligation of the left anterior descending (LAD) artery as described previously [3, 25]. In brief, mice (8–12 weeks of age, 20–25 g) were randomized into four groups: the sham group (sham), MI group, MI + MSC group, and MI + FNDC5-MSC group. C57BL/6 mice were anesthetized with isoflurane and mechanically ventilated. The heart was exposed by left thoracotomy. Then, LAD artery was permanently ligated with a 6-0 silk suture. When the anterior wall of the left ventricle (LV) turned pale and characteristic electrocardiographic (ECG) changes were recorded, success of the ligation was confirmed. At last, the chest and skin were sealed, and mice were placed on the ventilator until they woke up. Mice in the sham-operated control group underwent the same operation except that the suture below the left coronary artery was not ligated. Completely randomized design and blinding were adopted in animal experiments.

### BM-MSC transplantation

As described previously [3, 21], BM-MSC transplantation was performed immediately after MI. In brief, BM-MSCs were collected and randomly divided into the different groups separately. The suspended cells (1 × 10^6^) were injected directly into the peri-infarcted areas [at 2 sites near the peri-infarct zone (medial and lateral zones)] by a Hamilton syringe with a 29-gauge needle (in 20 mice in every group).

### In vivo BLI of engrafted BM-MSCs

BLI was performed to track transplanted BM-MSCs using an IVIS® Kinetic system (Caliper, Hopkinton, MA, USA) [1]. After intraperitoneal injection with d-luciferin (375 mg/kg body weight), recipient mice were anesthetized with isoflurane and imaged for 10 min on days 1, 7, 14, 21, and 28 until sacrificed. Peak signals (photons/s/cm^2^/sr) from a fixed region of interest (ROI) were analyzed with Living Image® 4.0 software (Caliper, MA, USA). All these assays were performed in a blinded manner.

### Evaluation of BM-MSC engraftment

Five mice were sacrificed 2 weeks after MSC engraftment to determine the BM-MSC survival in ischemic myocardium [21]. Then, the hearts were harvested, rapidly (within a minute) fixed in 4% paraformaldehyde. Serial sections were prepared at 5 μm thickness. BM-MSCs were stained with an FITC-labeled anti-GFP antibody and 4,6-diamidino-2-phenylindole (DAPI) to improve the identification of GFP-fluorescence in the frozen sections. Meanwhile, cardiomyocytes were stained with an anti-cTnI antibody. Engraftment of MSCs was confirmed by identification of GFP expression with fluorescent microscopy. The numbers of GFP-positive cells and DAPI in each slide were calculated. The data were expressed as the percentage of GFP^+^/DAPI in 5 slides obtained from five frozen sections. All assays were performed in a blinded manner.

### Histological analysis of fibrosis and angiogenesis

As described previously [26], fast green/sirius red stain was performed to detect fibrosis in cardiac muscle in 4-week post-procedure. Fibrosis was evaluated by measuring the collagen area as a proportion of the total left ventricular area with Imaging Pro Plus software. Meanwhile, the capillary density was determined by CD31 immunohistochemistry. Vessels in the peri-infarct zone were counted in randomly chosen 5 high-power fields (HPFs, magnification × 400). The results are expressed as vessels per HPF.

### Echocardiographic measurements

Cardiac function was measured under anesthesia with 2% isoflurane by transthoracic echocardiography at baseline, 7 days, and weekly until sacrifice at 4-week post-operation with a 30-MHz transducer on a Vevo® 2100 ultrasound system (VisualSonics, CA, USA) [21, 25]. Briefly, mice were anesthetized (2% isoflurane and oxygen) and put in a supine position. M-mode images and grayscale two-dimensional parasternal short-axis images at the mid-papillary level of each mouse were recorded. Measurements were carried out offline by a single observer in a group-blinded manner. The left ventricular end-systolic diameter (LVESD) and end-diastolic diameter (LVEDD) were measured from M-mode images. Meanwhile, left ventricular end-systolic volume (LVESV) and left ventricular end-diastolic volume (LVEDV) were also measured to calculate left ventricular ejection fraction (LVEF) and fractional shortening (FS) with the following equations: LVEF = (LVEDV − LVESV)/LVEDV × 100% and LVFS = (LVEDD − LVESD)/LVEDD × 100%. All the echocardiographic measurements were performed for three times in a blinded manner.

### Determination of myocardial apoptosis

Myocardial apoptosis at 48 h after BM-MSC transplantation was determined by terminal-deoxynucleotidyl transferase mediated-dUTP nick-end labeling (TUNEL) assay as previously described [27]. In brief, apoptotic cell nuclei and 4,6-diamidino-2-phenylindole (DAPI) (Sigma) stained all cell nuclei. Additional staining was performed with a monoclonal antibody against Troponin I (cTnI, Santa Cruz) for the identification of myocardium. Sections were imaged using confocal microscope (Fluo-View-FV1000, Olympus, Japan). The total number of nuclei and the number of TUNEL-positive nuclei were determined in five random fields from the border zone of the infarct in each sample. Meanwhile, the percentage of apoptotic cells was calculated. All these assays were performed in a blinded manner.

### Western blot assay

Cells were collected and dissolved in protein lysis buffer (Sigma). Then, equivalent protein (50 μg/lane) was separated by electrophoresis on 12% SDS-PAGE gels at 120 V for 1.5 h. Furthermore, they were transferred to PVDF membrane by 300 mV electrophoresis for 1.5 h. Cellular membranes were subjected to immune blotting with primary antibodies overnight at the temperature 4 °C after blocked in 5% non-fat dry milk (BD Biosciences) with 1xTBST at room temperature for 1 h. After incubation with appropriate secondary antibodies binding to horseradish peroxidase, an enhanced chemiluminescene system (Amersham Bioscience) was used to visualize blot bands. Also, we determined densitometric analysis of Western blot with VisionWorks LS, version 6.7.1 [28]. The following antibodies were used: rabbit anti-mouse FNDC5 (1:1000, Cell Signaling Technology), CD81 (1:500, Abcam), CD63 (1:500, Abcam), Alix (1:500, Cell Signaling Technology), GAPDH (1:500, Abcam), and rabbit anti-mouse β-actin (1:1000, Abcam).

### Statistics analysis

Data analysis was performed with GraphPad Prism 5.0 (San Diego, CA, USA). All quantitative data are expressed as the mean ± SEM. The Shapiro-Wilk normality test was performed for checking normality of data distribution. Comparisons of parameters among three or more groups were performed with a one-way analysis of variance (ANOVA). Differences between 2 groups were compared by using Student’s *t* test. The Bonferroni testing was performed to determine the post hoc testing. *p* value < 0.05 was considered as statistical significance.

## Results

### Hypoxia increased apoptosis of BM-MSCs

**Fig. 1:**
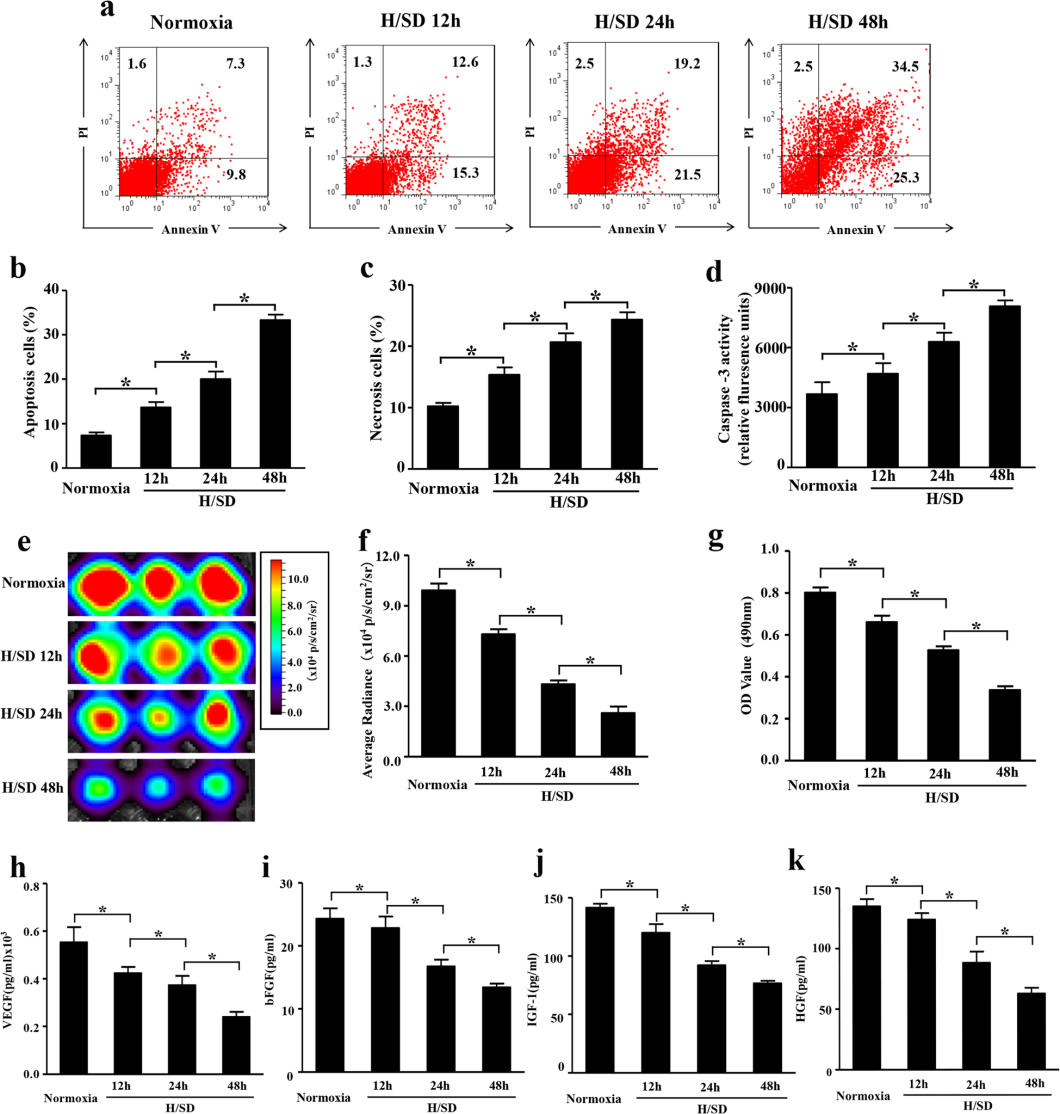
Hypoxia exposure increased apoptosis, reduced the viability, and restrained paracrine mechanism of BM-MSCs. **a** Representative results of the flow cytometry analyses in BM-MSCs under hypoxia for 12 h, 24 h, and 48 h. Viable cells, Annexin V^−^/PI^−^; early apoptosis, Annexin V^+^/PI^−^; late apoptosis, V^+^/PI^+^; necrotic, V^−^/PI^+^ (scale bars, 20 μm). **b**, **c** Quantification of the apoptotic BM-MSCs. **d** Histogram illustrating the caspase-3 enzymatic activity in MSCs^Fluc+GFP+^ after H/SD injury. **e** In vitro BLI results of BM-MSCs^Fluc+GFP+^ under normal conditions and after HS/D injury for 12 h, 24 h, and 48 h. **f** Representative quantification of BLI assays. **g** MTT assay indicated the effects of hypoxia (0 h, 12 h, 24 h, and 48 h) on viability of MSCs^Fluc+GFP+^. Representative ELISA assay illustrated the levels of vascular endothelial growth factor (VEGF) (**h**), basic fibroblast growth factor (bFGF) (**i**), insulin-like growth factor-1 (IGF-1) (**j**), and hepatocyte growth factor (HGF) (**k**) within MSCs under normal conditions and hypoxia for 12 h, 24 h, and 48 h. Data are expressed as the means ± SEM; *n* = 5; *p < 0.05

To analyze BM-MSC apoptosis induced by hypoxic stress, flow cytometry and caspase-3 activity assays were performed. Annexin V is considered a marker for early-stage apoptosis. Early-stage apoptotic cells will only take up Annexin V stain but will remain PI negative. The late-stage apoptotic and necrotic cells will be positive for both Annexin V and PI. The representative flow cytometry results shown in Fig. 1a–c indicated that the percentage of early-stage apoptotic cells under hypoxic condition significantly increased than that in normal condition (*p* < 0.05). In addition, compared with normal condition, cleaved caspase-3 was increased in hypoxia for 12 h. Meanwhile, with the increased hypoxia-time exposure, the expression of cleaved caspase-3 was elevated gradually (Fig. 1d, *p* < 0.05), which suggested that hypoxia promoted the cleaved caspase-3 expression level. These data suggested that hypoxic stress increased the apoptosis of BM-MSCs.

### Hypoxia exposure decreased BM-MSC viability

Representative in vitro BLI indicated a robust linear correlation between the cell number and average Fluc radiance (*r*^2^ = 0.98; Supplemental figure in the Data Supplement), suggesting that BLI of Fluc could be reliably used to monitor the viability of engrafted MSCs^Fluc+GFP+^ quantitatively in vivo. Furthermore, in vitro BLI displayed a remarkable decline of BLI signal intensity in MSCs^Fluc+GFP+^ after H/SD injury compared with normoxia (Fig. 1e, f, *p* < 0.05). Concurrently, MTT results also showed that hypoxia exposure impaired the viability of MSCs^Fluc+GFP+^ after H/SD injury (Fig. 1g, *p* < 0.05). Collectively, these data suggested that hypoxic stress decreased the viability of BM-MSCs.

### Hypoxic stress inhibited growth factor secretions in BM-MSCs

BM-MSCs are involved in cardiac repair and regeneration at least in part by paracrine effects. Therefore, we evaluated the effect of hypoxia on cytokine secretion in BM-MSCs. Results demonstrated that H/SD injury suppressed VEGF secretion compared with that in the normoxic group (Fig. 1h, *p* < 0.05). Also, the ELISA assay results illustrated that the bFGF, IGF-1, and HGF were restrained in BM-MSCs with hypoxic treatment (Fig. 1i–k, *p* < 0.05). Taken together, these results showed that H/SD injury contributed to the paracrine dysfunction of BM-MSCs.

### H/SD injury reduced expression of FNDC5 in BM-MSCs

**Fig. 2:**
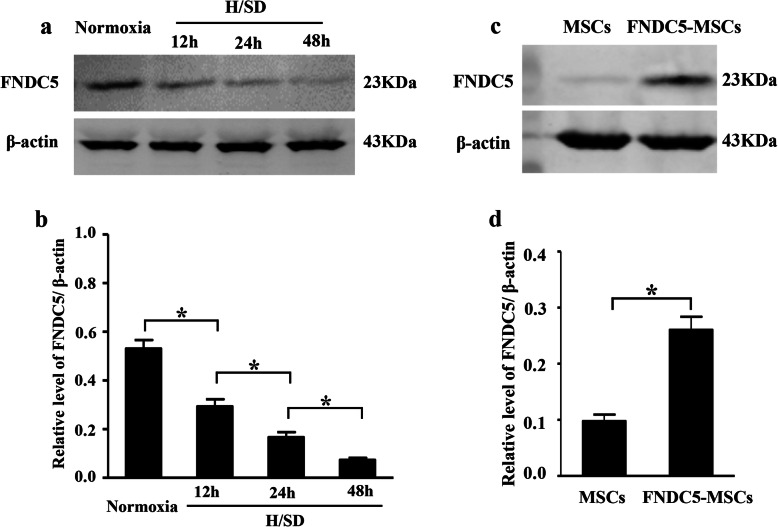
Hypoxia restrained the expression of FNDC5 of BM-MSCs. **a**, **c** Representative Western blot results in BM-MSCs. **b**, **d** Semi-quantification of FNDC5 expression. Data are expressed as the mean ± SEM; *n* = 5; *p < 0.05

To explore the effect of hypoxic stress on the protein expression of FNDC5, Western blot was performed. The representative results shown in Fig. 2a indicated that compared with the normoxic group, the protein expression level of FNDC5 was decreased in the hypoxia exposure group. Meanwhile, semi-quantitative analysis demonstrated that the expression of FNDC5 was decreased in hypoxia for 12 h compared with the normoxic group (*p* < 0.05, Fig. 2b). At the same time, the expression level of FNDC5 was restrained gradually after hypoxic exposure for 24 h and 48 h, respectively. In a word, these results showed that hypoxic injury suppressed the expression of FNDC5 in BM-MSCs.

To further increase the expression of FNDC5 in hypoxic BM-MSCs, FNDC5 was overexpressed by transfection. Results suggested that expression level of FNDC5 was promoted in FNDC5-MSCs (*p* < 0.05, Fig. 2c, d).

### FNDC5/irisin ameliorated BM-MSC apoptosis induced by H/SD injury

**Fig. 3:**
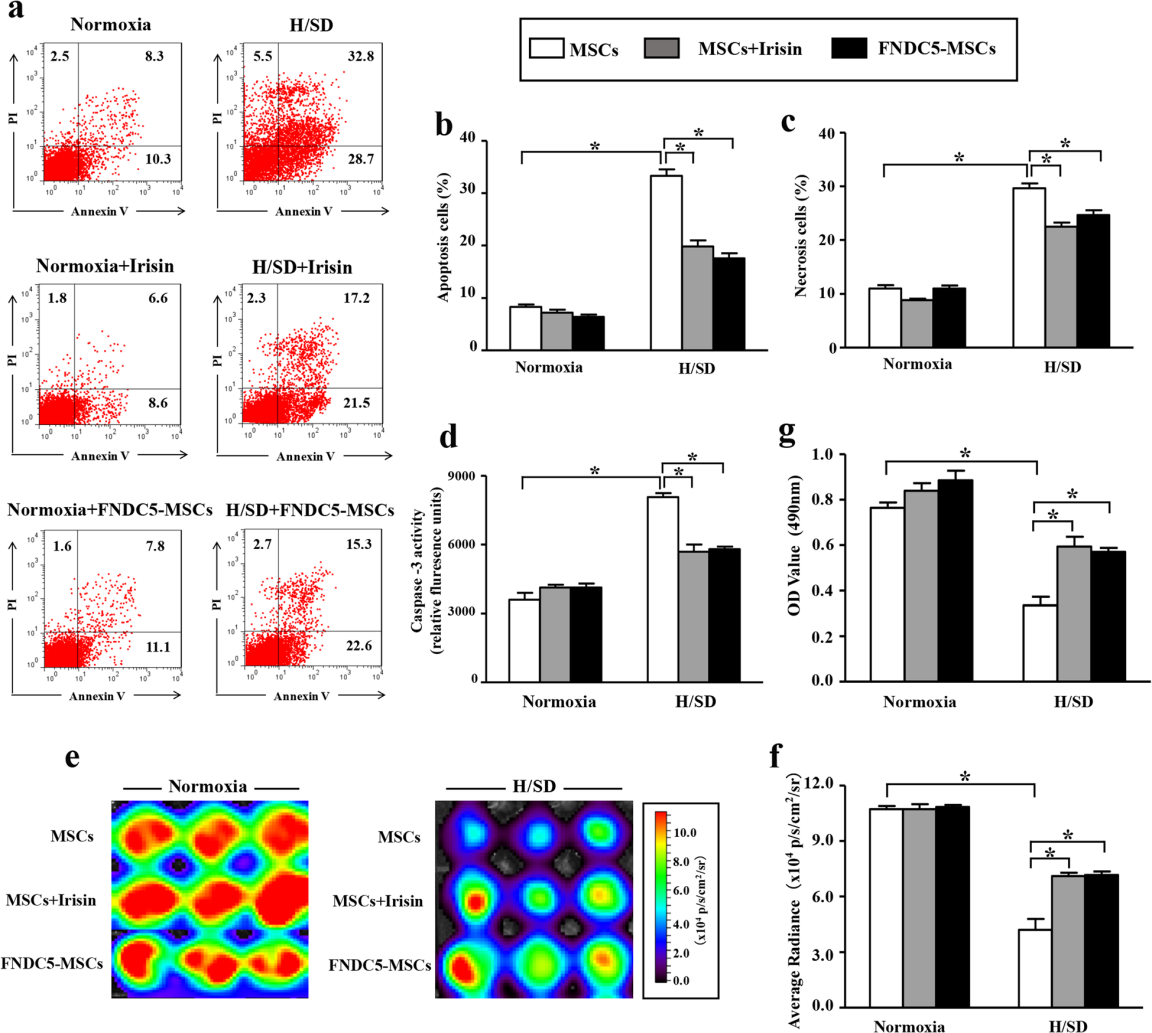
FNDC5/irisin reduced the increased apoptosis of BM-MSCs with hypoxia for 48 h. **a** Results of the flow cytometry analyses in BM-MSCs under normoxia, hypoxia for 48 h, H/SD + FNDC5-MSCs, and HS/D + irisin (scale bars, 20 μm). **b**, **c** Representative quantification of the apoptotic BM-MSCs under different treatments. **d** Histogram illustrating the caspase-3 enzymatic activity in BM-MSCs after various treatments. **e** Representative in vitro BLI results of MSCs^Fluc+GFP+^ with normoxia, hypoxia for 48 h, H/SD + FNDC5-MSCs, and HS/D + irisin. **f** Representative quantification of BLI assays in different groups. **g** MTT assay demonstrated the effects of FNDC5-OV/irisin on the viability of MSCs^Fluc+GFP+^. Data are expressed as the means ± SEM; *n* = 5; *p < 0.05

Flow cytometry and caspase-3 activity assays were performed for confirming whether FNDC5/irisin plays a protective role in hypoxia-induced apoptosis in BM-MSCs. The flow cytometry results illustrated that the percentage of early-stage apoptotic BM-MSCs under hypoxia for 48 h significantly increased than that in normal condition (Fig. 3a–c, *p* < 0.05). Also, the expression of cleaved caspase-3 was promoted under hypoxia for 48 h (Fig. 3d, *p* < 0.05). These data suggested that hypoxic stress enhanced the apoptosis of BM-MSCs. Interestingly, FNDC5-OV or irisin administration alleviated the increased apoptosis in hypoxia exposure for 48 h (Fig. 3a–d, *p* < 0.05).

### FNDC5/irisin alleviated the reduced viability of BM-MSCs induced by hypoxia

The in vitro BLI was performed to confirm the effect of FNDC5/irisin on cell viability. Representative results in Fig. 3e displayed that the BLI signal intensity in MSCs ^Fluc+GFP+^ in the FNDC5-OV group and MSCs + irisin group was significantly increased compared with that in the H/SD group (Fig. 3f, *p* < 0.05). Meanwhile, MTT results demonstrated that FNDC5-OV or irisin treatment promoted the impaired viability of MSCs^Fluc+GFP+^ under hypoxia condition for 48 h (Fig. 3g, *p* < 0.05). Collectively, these results suggested that FNDC5 or irisin played a protective role in the viability of BM-MSCs.

### FNDC5/irisin improved paracrine functions of hypoxic BM-MSCs

**Fig. 4:**
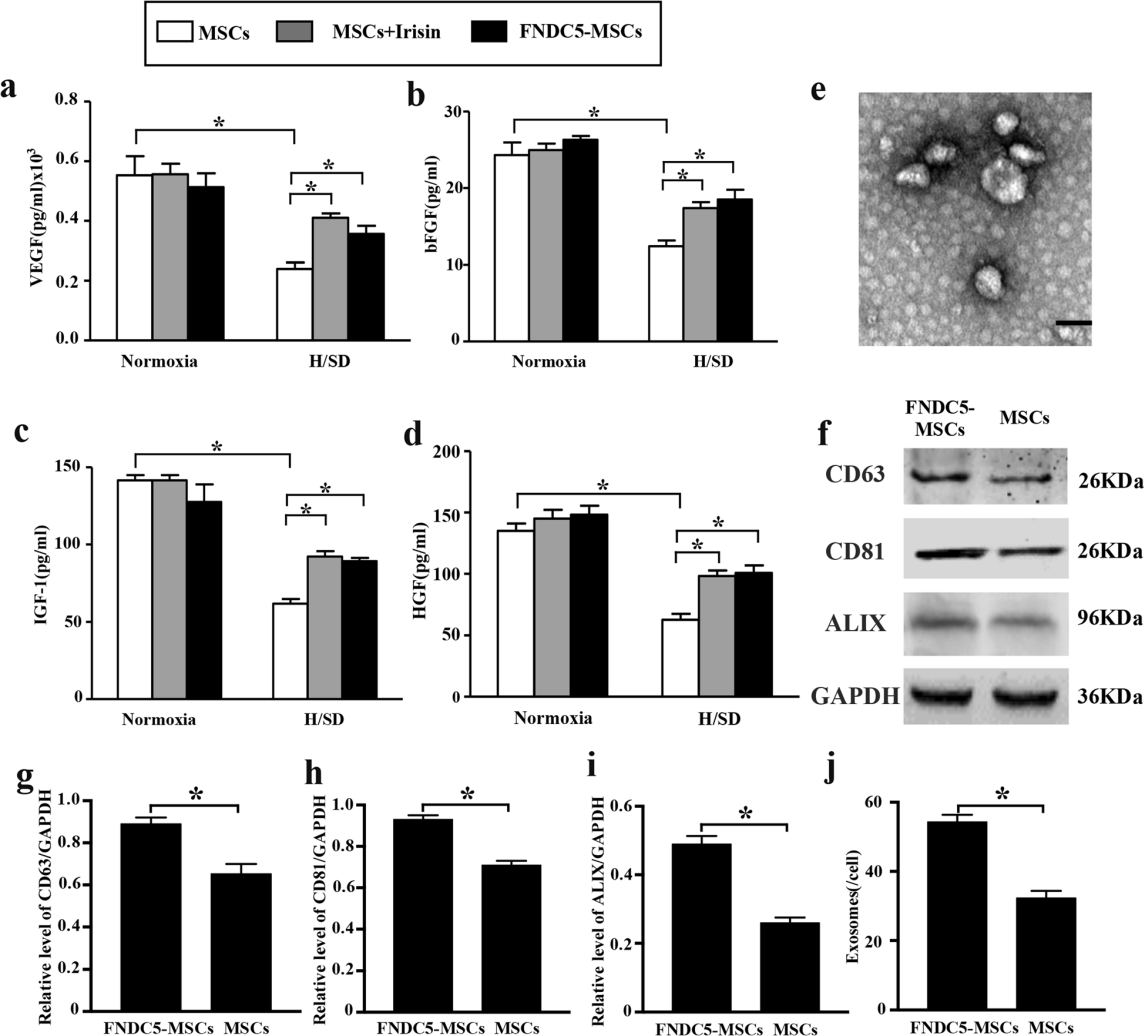
FNDC5-OV/irisin ameliorated the paracrine dysfunction of BM-MSCs under hypoxia for 48 h. Representative ELISA assay illustrated the levels of VEGF (**a**), bFGF (**b**), IGF-1 (**c**), and HGF (**d**) in BM-MSCs under various groups. **e** Transmission electron micrograph of exosomes deprived from BM-MSCs. Cup-shaped structures, 30–100 nm in size, were identified as exosomes. Scale bar, 200 nm. **f** Representative Western blot analysis for the exosome markers Alix, CD63, and CD81 in exosomes. Semi-quantification of CD63 (**g**), CD81 (**h**), and Alix (**i**) expression. **j** NTA showed the concentration of the isolated exosomes derived from BM-MSCs and FNDC5-MSCs (*n* = 5). Data are expressed as the mean ± SEM; *n* = 5; *p < 0.05

To investigate the effects of FNDC5/irisin on growth factor secretions in BM-MSCs, we performed ELISA assays. The representative results in Fig. 4a–d demonstrated that the secretions of VEGF bFGF, IGF-1, and HGF were increased after FNDC5-OV or irisin administration compared with that in hypoxia for 48 h (*p* < 0.05). At the same time, ultrastructure analysis demonstrated that BM-MSC exosomes had a cup-shaped membrane-bound vesicle with a diameter of approximately 100 nm (Fig. 4e). Western blot revealed that these exosomes expressed classical exosomal markers: CD81, CD63, and Alix (Fig. 4f). Notably, the expression levels of these exosomal markers were elevated in the FNDC5-MSC group compared with the MSC group (Fig. 4g–i, *p* < 0.05). In addition, as shown in Fig. 4j, the production of exosomes from FNDC5-MSCs was increased than that in the MSC group (*p* < 0.05). Taken together, these results illustrated that FNDC5/irisin ameliorated the paracrine dysfunction of BM-MSCs induced by hypoxia. Interestingly, FNDC5-OV promoted the secretion of exosomes in BM-MSCs.

### FNDC5 enhanced the retention effect of engrafted BM-MSCs

**Fig. 5:**
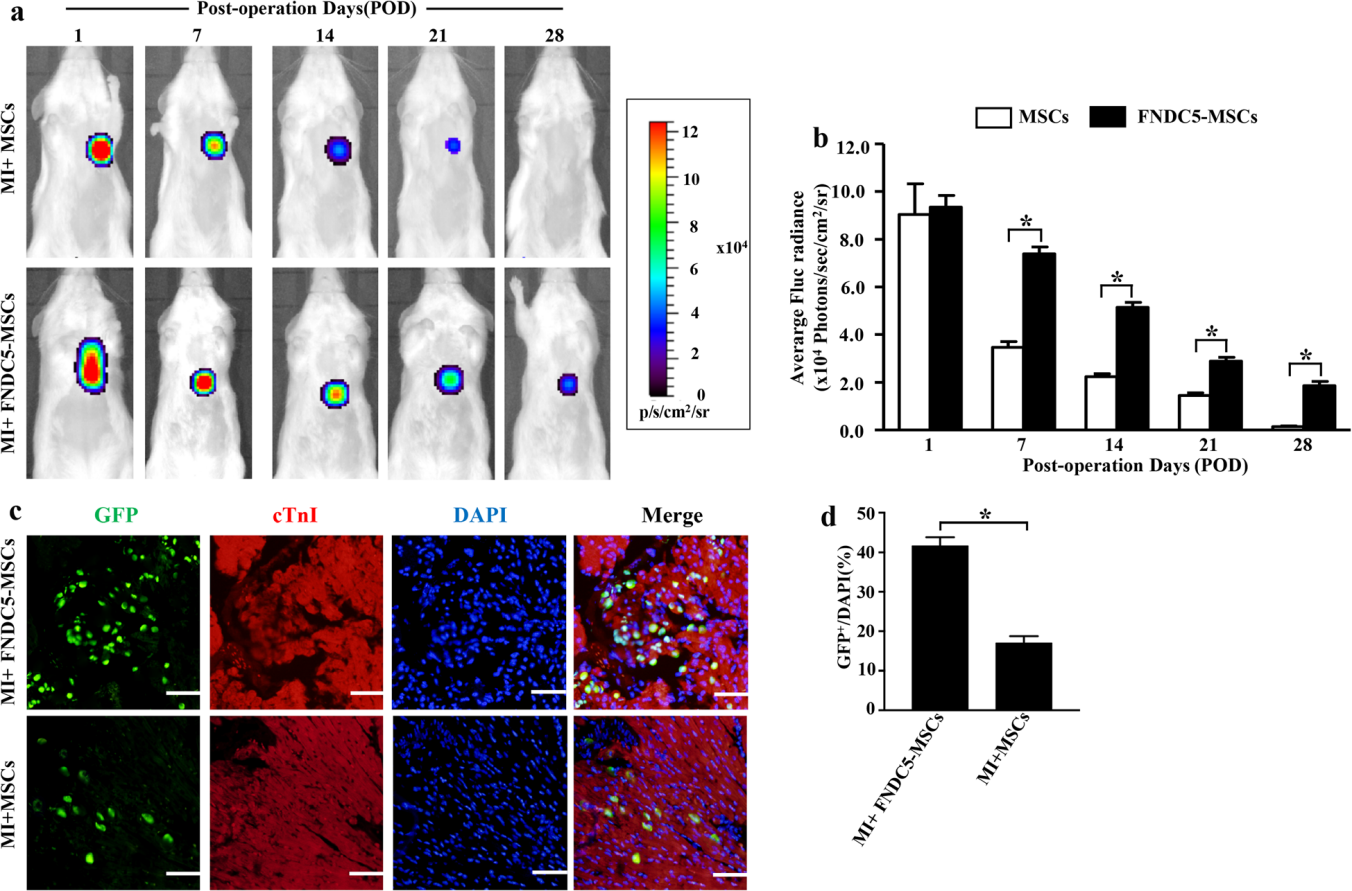
FNDC5 promoted the retention effect of engrafted BM-MSCs. **a** Longitudinal BLI spatiotemporally tracked MSCs^Fluc+GFP+^ survival in MI with preconditioned mesenchymal stem cells (MPCMSCs) (top row, *n* = 10) and FNDC5-MSCs preconditioned MSCs (MFPCMSCs) (second row, *n* = 10). Color scale bar values are in photons/s/cm^2^/sr. **b** Representative quantitative analysis of firefly luciferase (Fluc) optical signals on fixed regions of interest (ROI). **c** Representative confocal laser microscopic images of BM-MSCs (GFP, green fluorescence), cardiomyocytes (cTn I, red florescence), and DAPI (blue fluorescence) at 2 weeks after engraftment (scale bar:50 μm). **d** Quantitative analysis of the ratio of GFP/DAPI. Data are expressed as means ± SEM; *n* = 5; *p < 0.05

Longitudinal BLI was performed for determining the retention of BM-MSCs transplanted into infarcted hearts. Representative BLI results in Fig. 5a showed a progressive decay of BLI signal within 4 weeks after engraftment in the BM-MSC group. By contrast, FNDC5-OV promoted the retention of engrafted MSCs^Fluc+GFP+^ (Fig. 5b, *p* < 0.05). As shown in Fig. 5c, the GFP-positive cells were more frequently observed in MI mice administered with FNDC5-MSCs. Furthermore, the ratio of GFP^+^/DAPI in the MI + FNDC5-MSC group was 41.67 ± 2.18%, significantly higher than that in the MI + MSC group (17.00 ± 1.73%, *p* < 0.05, Fig. 5d). Collectively, FNDC5 overexpression improved the retention effect after MSC engraftment.

### FNDC5-MSCs reduced fibrosis, elevated pro-angiogenic effect, and improved cardiac function after MI

**Fig. 6:**
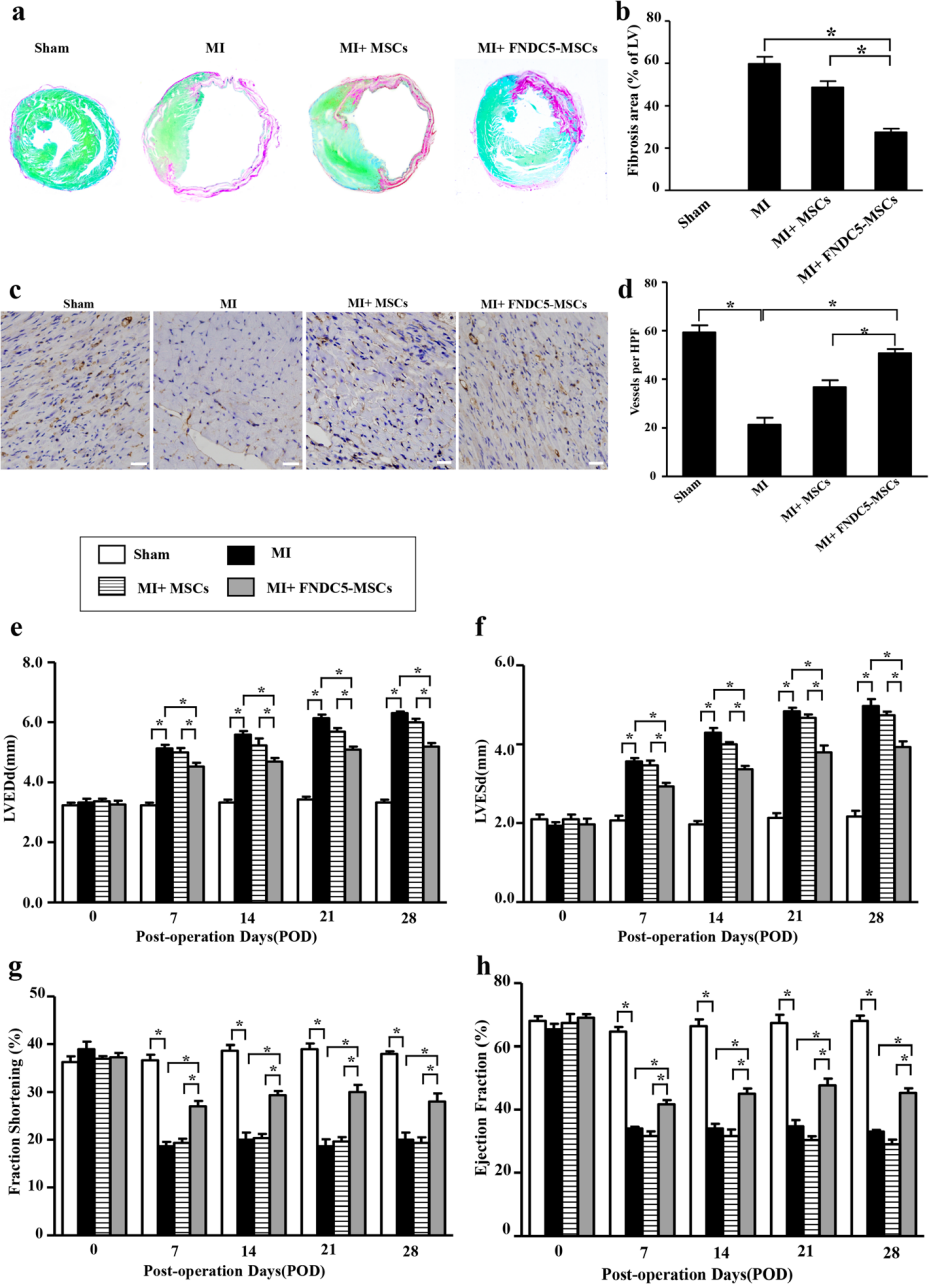
Evaluation of fibrosis, pro-angiogenic effect, and heart function after different groups. **a** Masson’s trichrome staining indicated left ventricular fibrosis 4 weeks after various treatments (magnification × 4). **b** Representative quantitative analysis of the fibrotic area. **c** Capillaries in the infarct border zone were determined by immunohistochemical staining for CD31-positive cells in different groups (scale bars = 50 μm). **d** Representative capillaries in the infarct border zone. Histograms illustrating the heart function parameters: left ventricular end-diastolic diameter (LVEDD, **e**), left ventricular end-systolic diameter (LVESD, **f**), left ventricular fractional shortening (**g**), and left ventricular ejection fraction (**h**). Data are expressed as means ± SEM; *n* = 5; *p < 0.05

To study the effects of FNDC5 on the therapeutic efficiency of transplanted BM-MSCs, sirius red/fast green and CD31 staining were performed to evaluate the fibrosis and angiogenic effect. Furthermore, echocardiography analysis was performed to evaluate the heart function. As shown in Fig. 6a, BM-MSCs decreased cardiac fibrosis after MI. However, there was no significant difference between the MI group and MI + BM-MSC group statistically. Interestingly, FNDC5-MSCs significantly reduced myocardial fibrosis compared with the MI and MI + BM-MSC groups (Fig. 6a, b, *p* < 0.05). Consistently, the number of capillaries in myocardial tissue was evaluated by CD31 staining. The representative immunohistochemistry results demonstrated that the number of capillaries was reduced in the MI group and in the MI + BM-MSC group. And there was no statistical difference between the MI group and MI + BM-MSC group. Interestingly, FNDC5-MSCs increased the number of capillaries compared with that of the MI + BM-MSC group (Fig. 6c, d, *p* < 0.05). In addition, echocardiographic analysis revealed that the baseline parameters were similar in all groups. However, the left ventricle (LV) dimensions (LVEDD and LVESD) were increased after MI. Meanwhile, the LV dimensions were decreased in the FNDC5-MSC group compared with the MI and MI + BM-MSC groups (Fig. 6e, f, *p* < 0.05). Moreover, transplantation of BM-MSCs also manifested a trend towards improvement of cardiac performance over the 4 weeks after MI. Interestingly, the apparent benefit of BM-MSC transplantation was significantly promoted by FNDC5-MSCs (Fig. 6g, h, *p* < 0.05). Taken together, these data suggested that the therapeutic benefits of BM-MSC transplantation after MI are enhanced by FNDC5.

### FNDC5-MSCs decreased cardiomyocyte apoptosis after MI

**Fig. 7:**
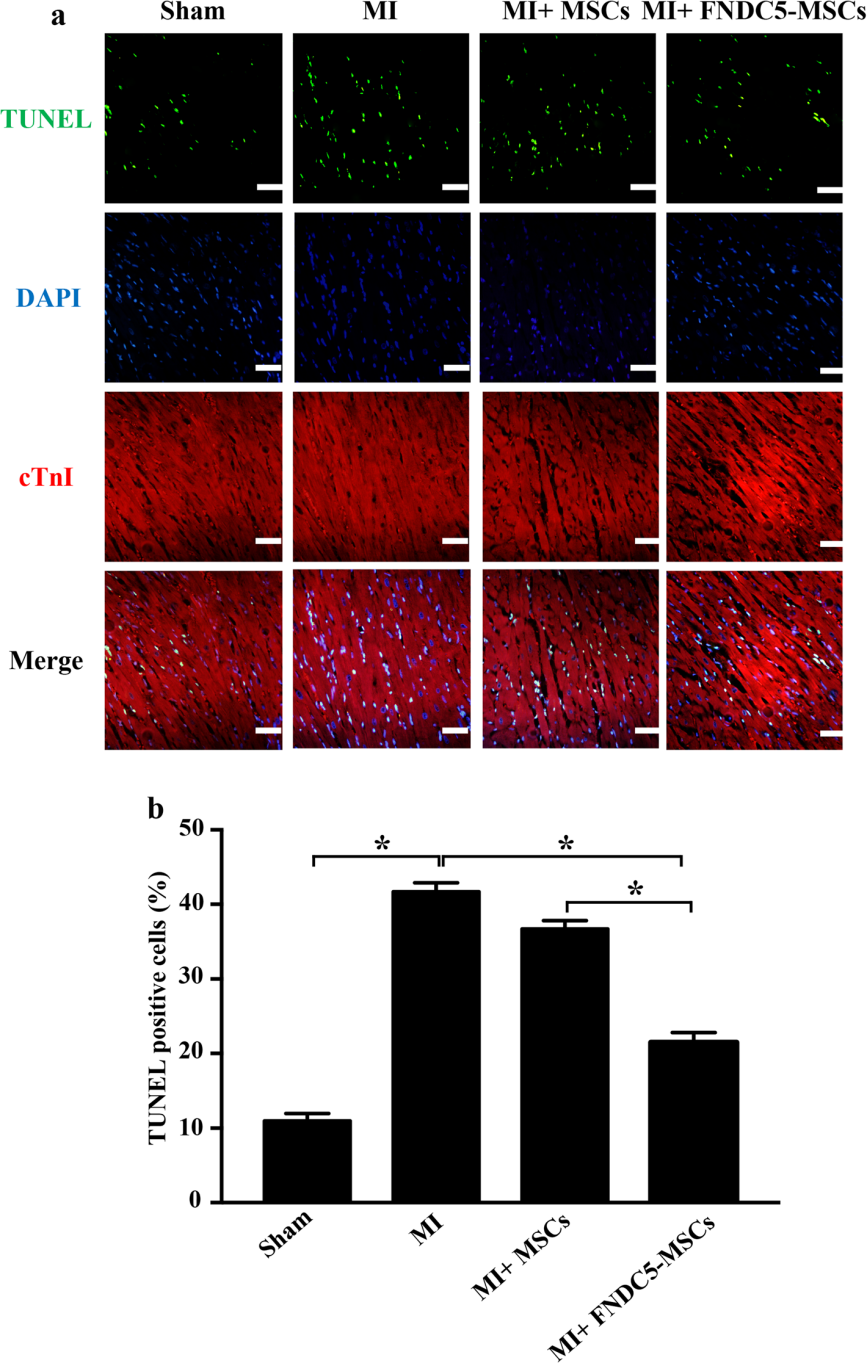
Effects of FNDC5-MSCs on cardiomyocyte apoptosis. **a** Confocal microscopy showed that TUNEL-positive cardiomyocytes were more frequently observed in the MI group compared with the MI + FNDC5-MSC group. Apoptotic nuclei were identified as TUNEL positive (green fluorescent) and total nuclei by DAPI counterstaining (blue fluorescent). Myocardium was stained using a monoclonal antibody against cTnI (red fluorescent). Scale bar represents 20 μm. **b** Representative TUNEL-positive apoptotic cells. Data are expressed as means ± SEM; *n* = 5; *p < 0.05

TUNEL assay was performed to confirm the protective effect of FNDC5-MSCs on the apoptosis of cardiomyocytes after MI. As shown by representative photomicrograph (Fig. 7a), apoptotic cardiomyocytes, which were manifested by TUNEL positivity (in green), were more frequently observed in the MI group than that in the sham group (41.70 ± 0.69% vs. 10.97 ± 0.58%; *p* < 0.05, Fig. 7b). Furthermore, the apoptosis-positive cells were remarkably reduced in the MI + FNDC5-MSC group, compared with that in the MI and MI + BM-MSC groups (41.70 ± 0.69% vs. 21.47 ± 0.78%; 36.67 ± 0.66% vs. 21.47 ± 0.78%, *p* < 0.05, Fig. 7b). Taken together, our data indicated that FNDC5-MSCs decreased the apoptosis of cardiomyocytes after MI.

## Discussion

**Fig. 8:**
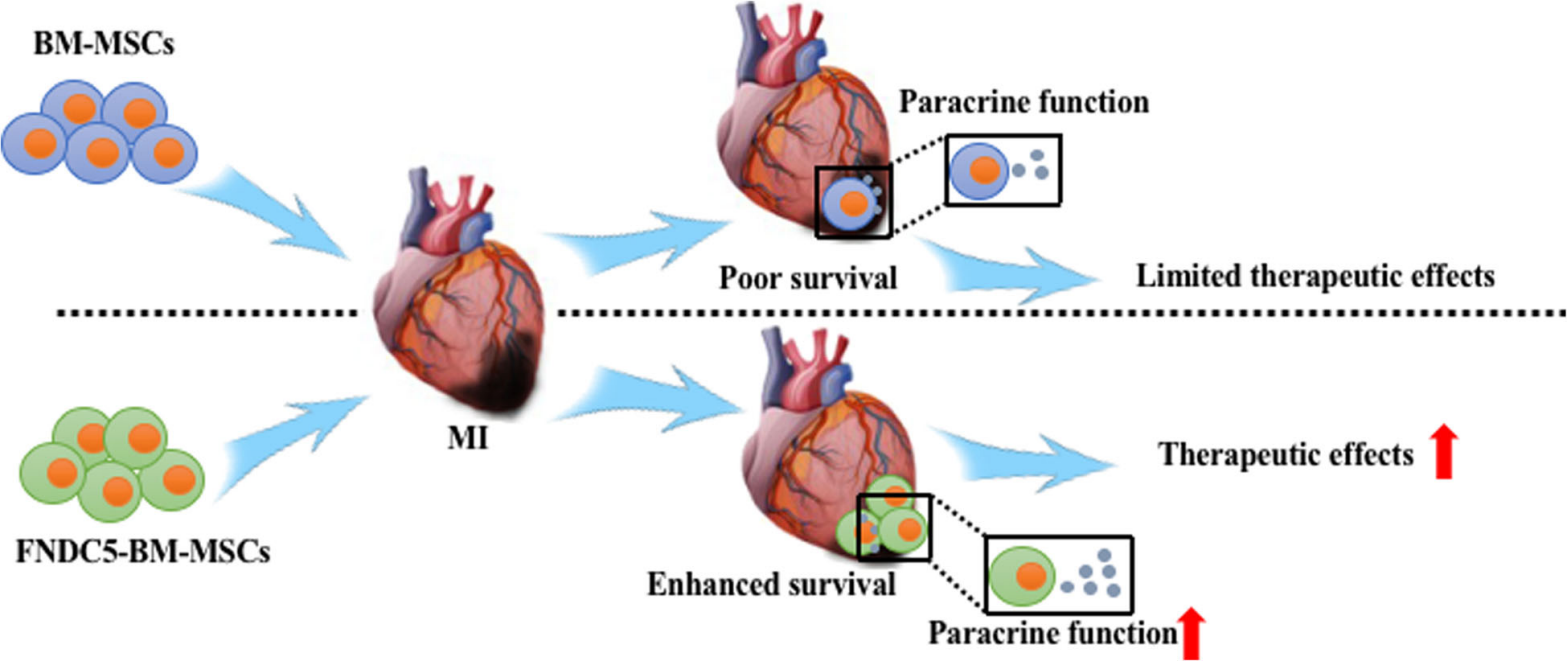
Proposed hypothesis of FNDC5/irisin improving the therapeutic efficacy of BM-MSCs for MI. FNDC5/irisin may protect against promoted apoptosis and paracrine dysfunction of BM-MSCs induced by hypoxia. Furthermore, FNDC5/irisin may contribute to enhance the low survival of engrafted BM-MSCs and ameliorate cardiac dysfunction after MI

In the present study, our data indicated that H/SD injury inhibited the protein expression levels of FNDC5 in BM-MSCs. Meanwhile, hypoxic stress involved in BM-MSC paracrine dysfunction increased cell apoptosis and viability. Furthermore, FNDC5-OV and irisin attenuated the BM-MSC injury induced by hypoxia exposure. Interestingly, FNDC5-OV elevated the survival of BM-MSCs after transplantation. Furthermore, FNDC5-MSC transplantation decreased the apoptosis and fibrosis of infarcted myocardium, increased capillary density, and finally improved cardiac function. Collectively, our results suggested that FNDC5/irisin may be a proposed optimized strategy of BM-MSC therapy for MI (Fig. 8).

MSC therapy for MI has achieved a promising stage, but studies also demonstrated that only marginal improvements in cardiac function were observed after the engraftment of MSCs into infarcted heart tissue [26, 29]. The stem cell therapy for clinical application is limited, mainly as a result of the reduced viability of implanted MSCs. Our previous study has revealed a high level of MSC apoptosis between days 3 and 7 after implantation [21]. In the present study, we tracked the transplanted BM-MSCs by BLI and also found similar acute cell death within 1 week after transplantation. Moreover, engraftment with BM-MSCs alone failed to prevent cardiomyocyte death and improve cardiac dysfunction significantly, which was consistent with the results of Zhang et al. [26, 30]. Lack of nutrients and oxygen in ischemic myocardium has been considered as the major factors which contributed to the decreased viability of MSCs after transplantation [31]. Therefore, H/SD in vitro cellular model was performed to mimic the in vivo ischemic environment [32]. Concurrently, our results revealed that H/SD injury reduced cell survival and caused paracrine dysfunction of BM-MSCs [12]. Thus, promoting the low viability of BM-MSCs under hypoxic conditions after transplantation is crucial for elevating the efficiency of cellular therapy.

FNDC5 was recognized as a transmembrane protein with a fibronectin III domain and a carboxy-terminal domain located in the cytoplasm [33]. Irisin is the extracellular domain of FNDC5, which is cleaved by enzyme [14, 34]. Meanwhile, irisin has been proved as a multifunctional peptide that could bind to an unidentified receptor to play various effects [15, 16]. Fatouros et al. [35] found that irisin was secreted by skeletal muscles. However, Yu et al. [36] indicated that the expression of irisin also increased in the heart of hypertrophic mice. They found that angiotensin II-induced myocardial cell hypertrophy was attenuated after administration of irisin in vitro, suggesting irisin also could be derived from the heart and had a cardioprotective effect on cardiomyocyte dysfunction. In the current study, we found for the first time that hypoxia exposure decreased the protein expression level of FNDC5 in BM-MSCs. Moreover, we also found that irisin/FNDC5 improved survivals of BM-MSCs under hypoxic condition. Recently, genetic modification has attracted great attention as a potential approach to improve the low viability of cells [8, 37–39]. For this reason, we upregulated the FNDC5 expression in BM-MSCs and transplanted FNDC5-MSCs into the infarcted heart. Interestingly, our BLI and immunofluorescence results suggested the FNDC5 overexpression enhanced the functional survival of BM-MSCs. Furthermore, FNDC5-MSCs significantly reduced fibrosis and apoptosis of cardiomyocytes, with improved cardiac function after MI.

At present, paracrine effects have been heavily implicated in MSC-based improved cardiac dysfunction after MI [40]. Our previous study indicated that BM-MSCs secreted various bioactive factors, such as VEGF, bFGF, HGF, and IGF-1 [1]. In the current study, we found that the paracrine secretion effects of donor BM-MSCs were suppressed by hypoxic stimuli while were dramatically enhanced after irisin or FNDC5-MSC treatment, suggesting that irisin or FNDC5 could play a cardioprotective role through paracrine secretion effects of BM-MSCs. Recently, exosomes from BM-MSCs have emerged as a potential strategy for MI [6, 41]. Therefore, we further investigated the effects of FNDC5 on exosomes derived from BM-MSCs. Interestingly, we observed for the first time that FNDC5 promoted secretions of exosomes in BM-MSCs. Taken together, these data suggested that FNDC5/irisin may be a potential optimizing target for BM-MSC-based cellular therapy for MI.

Although the present study bears some clinical relevance, there are several limitations. Firstly, the in vivo H/SD cellular model cannot fully mimic the in vivo ischemic environment. Secondly, the number of animal experimental samples was small. Finally, the detailed molecular mechanism by which FNDC5/irisin exerted protective effects on hypoxic MSCs was not clarified completely. Therefore, research on specific mechanisms warrants further dedicated investigation.

## Conclusions

Collectively, our in vitro and in vivo data embodied the FNDC5/irisin-ameliorated hypoxia injury of BM-MSCs. Furthermore, gene modified with FNDC5 overexpression increased the survival of BM-MSCs after engraftment and improved the cardiac functions after MI, suggesting that FNDC5/irisin may be a potential optimizing target for BM-MSC-based cellular therapy for MI.

## Supplementary information

**Additional file 1: Supplemental figure.** Representative in vivo BLI showed a linear relationship between cells number and Fluc reporter gene activity.

## Data Availability

The data sets supporting the results of this article are included within the article and its additional files.

## Abbreviations

FNDC5: Fibronectin type III domain-containing protein 5
BM-MSCs: Bone marrow mesenchymal stem cells
MI: Myocardial infarction
H/SD: Hypoxia and serum deprivation
Fluc: Firefly luciferase
eGFP: Enhanced green fluorescence protein
DMEM: Dulbecco’s modified Eagle’s medium
FBS: Fetal bovine serum
MTT: 3-(4,5-Dimethylthiazol-2-yl)-2,5-diphenyltetrazolium bromide
DMSO: Dimethyl sulphoxide
OD: Optical density
BLI: Bioluminescence imaging
ELISA: Enzyme-linked immunosorbent assay
VEGF: Vascular endothelial growth factor
bFGF: Basic fibroblast growth factor
IGF: Insulin-like growth factor
HGF: Hepatocyte growth factor
LAD: Left anterior descending
LV: Left ventricle
ECG: Electrocardiography
ROI: Region of interest
HPFs: High-power fields
LVESD: Left ventricular end-systolic diameter
LVEDD: Left ventricular end-diastolic diameter
LVESV: Left ventricular end-systolic volume
LVEDV: Left ventricular end-diastolic volume
LVEF: Left ventricular ejection fraction
FS: Fractional shortening
TUNEL: Terminal-deoxynucleotidyl transferase mediated-dUTP nick-end labeling
DAPI: 4,6-Diamidino-2-phenylindole
