# Supplementary figures and images for: FNDC5/irisin improves the therapeutic efficacy of bone marrow-derived mesenchymal stem cells for myocardial infarction
Source: Stem Cell Res Ther. 2020 Jun 10;11:228. doi: 10.1186/s13287-020-01746-z (PMC7288492; doi:10.1186/s13287-020-01746-z)

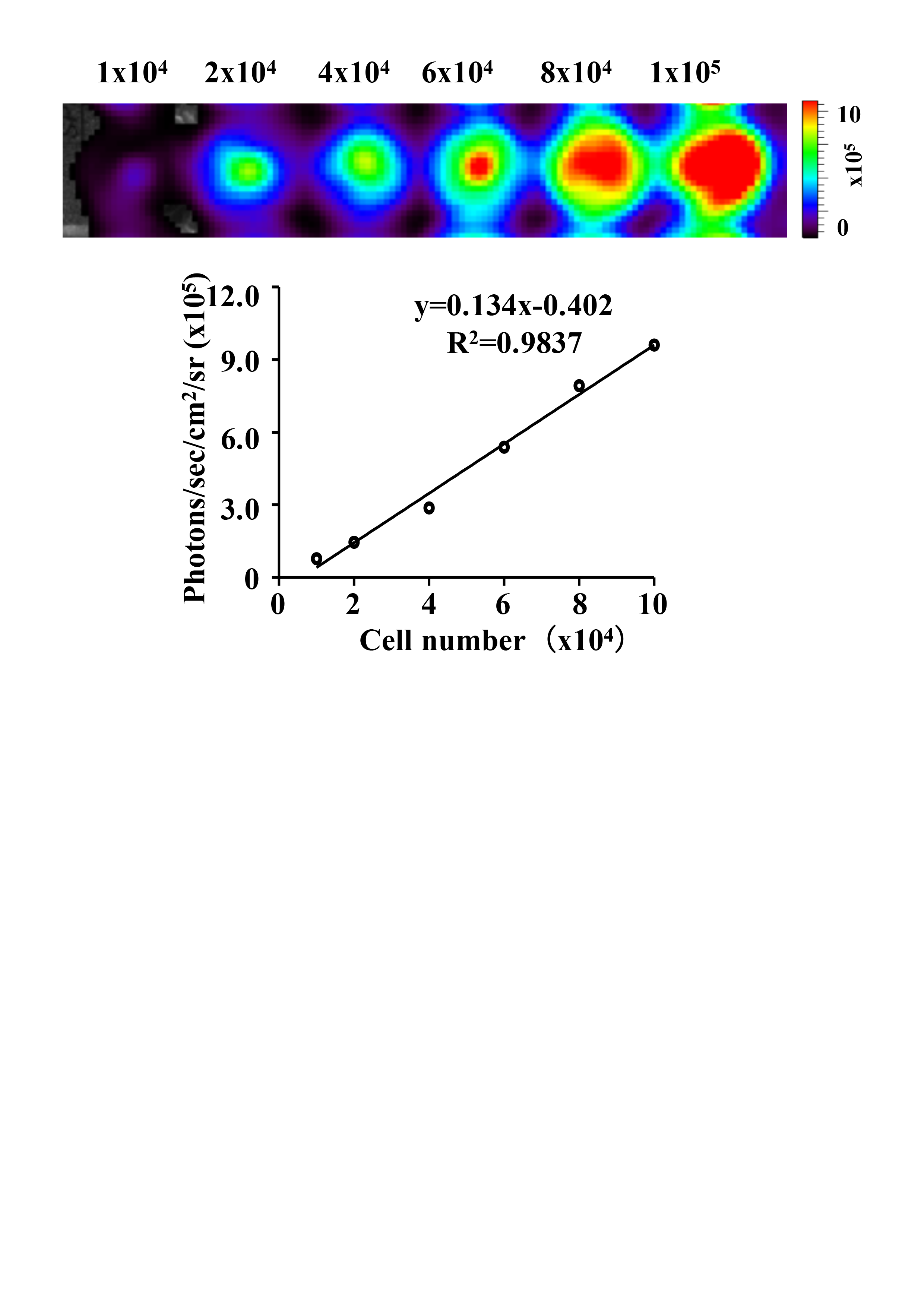

Supplement: Supplementary file 1 — Additional file 1: Supplemental figure. Representative in vivo BLI showed a linear relationship between cells number and Fluc reporter gene activity. [file 13287_2020_1746_MOESM1_ESM.tif]
